# The Tax Incidence and Tax Pass-Through of Smokeless Tobacco in the US

**DOI:** 10.3390/ijerph21111465

**Published:** 2024-11-02

**Authors:** Yanyun He, Qian Yang, Ce Shang

**Affiliations:** 1 Center for Tobacco Research, The Ohio State University Wexner Medical Center, 3650 Olentangy River Rd, Columbus, OH 43214, USA; qian.yang@osumc.edu (Q.Y.); ce.shang@osumc.edu (C.S.); 2 Department of Internal Medicine, Medical Oncology Division, The Ohio State University, Columbus, OH 43201, USA

**Keywords:** smokeless tobacco, tax incidence, tax pass-through

## Abstract

Background: States adopt different tax bases for smokeless tobacco (SLT), making tax incidence on SLT not directly comparable across states. In addition, how taxes are passed through to SLT prices among states that impose specific taxes, and whether the pass-through rates for SLT are affected by the uptake and evolution of e-cigarettes, is unknown. Objective: This study will calculate the tax incidence on SLT and investigate how SLT taxes are passed to prices at the 25th, 50th, and 75th percentile levels, as well as whether these pass-through rates vary by e-cigarette uptake and evolution. Methods: We regressed SLT prices on specific taxes using ordinary least square regressions while controlling for state-, year-, and quarter-fixed effects. We then tested the difference in tax pass-through rates by different periods. Findings: The average tax incidence on chewing tobacco, moist snuff, dry snuff, and snus was 22%, 22%, 23%, and 20%, respectively. For moist snuff, taxes were fully passed to prices at the 25th and 50th percentiles (rate = 1.01, *p* < 0.001) and overly passed to prices at the 75th percentile (rate = 1.25, *p* < 0.001). The e-cigarette uptake and evolution significantly raised taxes by 13 cents and 14 cents per ounce, respectively, for moist snuff at the 75th percentile prices (*p* < 0.05). Conclusions: If harm is considered a criterion for taxing tobacco products, the tax incidence on SLT could be further increased. Considering that lower-priced SLT have lower tax pass-through rates, price promotion restrictions and minimum pricing laws may be needed to increase the cost of lower-priced products. Additionally, we observed that tobacco companies tended to increase tax pass-through for premium SLT products as e-cigarettes gained popularity, which may indicate a strategic response to shifting consumer preferences.

## 1. Introduction

Smokeless tobacco (SLT) encompasses various non-combustible tobacco forms, including chewing tobacco, moist snuff, dry snuff, snus, and dissolvable products. In 2021, 2.1% of adults in the United States (US) used SLT every day or some days [1]. SLT use among high school students is comparable to cigarette smoking, with 1.5% and 1.9% reporting the past 30-day use of SLT and cigarettes in 2023 [2]. SLT use leads to a series of adverse health consequences, such as cancer of the mouth, esophagus, and pancreas [3,4]. There are also well-documented disparities in SLT use—rural populations are more likely to use SLT than urban populations, especially those living in Appalachian areas [1,2,5,6,7]. In 2022, moist snuff accounted for 90.7% of total SLT sales, underscoring its position as the most favored product within the SLT category [8].

Tobacco excise taxes are powerful public health tools to regulate smokeless tobacco use, further reducing adverse health consequences resulting from SLT use [9,10]. Leveraging taxes on SLT may also serve as a source of revenue that can be further spent on promoting health outcomes, making them a popular policy widely adopted by federal, state, and sometimes local authorities [11].

Despite the implementation of SLT taxes at the federal level and by all US states, their approaches to SLT taxation significantly vary, with some states using specific taxes based on product weight, while others employ *ad valorem* taxes as a percentage of retail, wholesale, or manufacturer prices. The federal taxes for SLT are based on weight, with the present tax rate set at 50.3 cents per pound for chewing tobacco and USD 1.51 per pound for other forms of SLT. This divergence in tax bases makes direct comparisons challenging. Moreover, some states impose differential tax bases or rates for different SLT forms, further complicating the evaluation and measurement of SLT taxes.

Considering the complexity, the tax incidence or burden for SLT is needed to measure and compare the magnitude of taxes across different bases and products. Such incidence measures have been developed for e-cigarettes (ECs) in the US for comparison and adopted by the World Health Organization (WHO) to guide the implementation of cigarette taxes in many countries with different tax bases (e.g., the WHO suggests that the tax incidence among retail prices should be at least 75%) [12,13]. However, the tax incidence for SLT products in different US states has not been explored yet in the existing literature, leaving a significant knowledge gap for policymakers when they design SLT taxes. In addition, the emergence of tobacco or nicotine options, such as oral nicotine pouches and ECs, prompted policymakers to consider taxing different products relative to their harms, making tax incidence particularly valuable because it allows for a direct comparison of taxation across products with a wide range of attributes and prices.

Another gap in the existing literature is the tax pass-through rate for SLT, which has not been studied in prior research. To increase prices by the amount of taxes, taxes need to be fully passed to prices. As a result, the tax pass-through rate determines whether the tax policy will be effective in changing behaviors. A number of studies have explored the tax pass-through rates for cigarettes and ECs [14,15,16,17]. However, a similar analysis has not been performed for SLT.

Moreover, since the introduction of ECs in the US in 2007, the US nicotine and tobacco marketplace has undergone significant changes. The EC use prevalence started to skyrocket after 2012. By 2014, ECs had replaced cigarettes as the most used tobacco product among youth [18]. Notably, nicotine salt-based ECs entered the market in 2015, with sales of JUUL pods experiencing an unprecedented 641% surge from 2.2 million in 2016 to 16.2 million in 2017 [19]. The increasing competition between different nicotine or tobacco products has changed the industry’s behaviors, including how they shift cigarette excise taxes to prices as the EC market evolves and merges with the cigarette market [14,20]. However, it is unknown how the tax pass-through rates for SLT have changed in response to EC uptake and evolution, especially when many SLT brands are owned by tobacco companies that also sell cigarettes and ECs [21].

To address the identified gaps in research, including (1) the lack of a standardized SLT tax measure, (2) the lack of evidence on SLT tax pass-through rates to prices, and (3) the lack of assessment of SLT tax pass-through trends in the era of emerging nicotine products, we first calculated tax incidence on SLT (the percentage of excise taxes among retail prices) for all states. Then, we examined the tax pass-through rate for states that impose specific taxes on SLT at different price levels (25th, 50th, and 75th percentile prices). We also tested how the tax pass-through rate to prices varied by different periods, including the pre-EC uptake era (2006–2011), the EC uptake era (2012–2016), and the evolution of the nicotine salt-based EC era (2017 and later). The implications of our findings extend to informing the design and refinement of the SLT taxation system, offering policymakers a nuanced understanding of existing SLT tax policies through comparisons across states.

## 2. Data and Methods

### 2.1. Data Sources for SLT Excise Taxes and SLT Prices

We sourced the quarterly state excise taxes on SLT from the Centers for Disease Control and Prevention (CDC) State Tobacco Activities Tracking and Evaluation (STATE) System [22]. The federal SLT excise taxes were obtained from the Alcohol and Tobacco Tax and Trade Bureau [23]. The federal taxes on SLT are based solely on the weight of the product, which is a specific US dollar amount per pound. We re-scaled the federal taxes to a US dollar per ounce so that we could calculate the total amount of taxes.

We obtained the historical price data for each type of SLT from the Nielsen Retail Scanner Data (NRSD) provided by the Kilts Center for Marketing at the University of Chicago Booth School of Business. This comprehensive dataset captures relevant information, such as prices, sales volume, product name/type, store environment, and more. It covers 90 participating retail chains across all US markets and has collected sales information every week since 2006. The total number of retail stores ranged from 30,000 to 50,000 from 2006 to 2020. Since there are very few sales records for dissolvable products, we excluded them from our analysis. We calculated the average price and prices at the 25th, 50th, and 75th percentiles for chewing tobacco, moist snuff, dry snuff, and snus, and aggregated them to a quarterly level for each state for regression analysis.

The SLT excise taxes and prices were adjusted for inflation before entering the regression equation that examines the tax pass-through rates. We used the regional Consumer Price Index published by the US Bureau of Labor Statistics in constant 2010 US dollars [24].

### 2.2. Data Sources for State-Level Tobacco Control Policies

State-level tobacco control policies, including regulations such as smoke-free indoor air laws for both cigarettes and ECs, as well as minimum legal sales age (MLSA) laws for cigarettes and ECs, were obtained from the CDC STATE System [22]. We assigned a binary indicator variable to determine the presence or absence of smoking or vaping bans in private worksites, restaurants, and bars within each state. A value of 1 was applied if the state had implemented a ban, and 0 if no such ban was in place. Similarly, for MLSA laws, we assessed whether the state had set the minimum legal sales age at 21 for both cigarettes and ECs. If this requirement was met, the corresponding indicator variables were assigned a value of 1; otherwise, they were assigned a value of 0.

### 2.3. Methods

#### 2.3.1. Calculating the Tax Incidence

Tax incidence is defined as the taxes paid as a percentage of final retail prices. The production and distribution of SLT typically follow a three-stage process: manufacturing, wholesale distribution, and retail distribution. Each stage adds value and involves pricing adjustments that influence the final retail price and tax incidence. At the manufacturing stage, raw tobacco is processed and turned into various SLT products. The manufacturer sets the initial price, which will then be marked up as the product moves through the supply chain. After production, SLT products are sold to wholesale distributors. Wholesalers purchase the products from manufacturers and add a markup to cover their own costs. In the final stage, retailers purchase smokeless tobacco products from wholesalers and sell them to consumers. Retailers apply their own markup to the wholesale price to cover their costs. This is the price at which consumers buy the product. For states that impose specific taxes, we used the following formula to calculate the tax incidence:

$${Tax\;incidence}_{s,t}=\frac{{Specific\;taxes}_{s,t}}{{Price\_r}_{s,\;year1}}$$

where ${Tax\;incidence}_{s,t}$ denotes the tax incidence for state *s* at time *t*, and ${Specific\;taxes}_{s,t}$ denotes the specific taxes in state *s* at time *t*, expressed as the US dollar amount per oz of SLT. ${Price\_r}_{year1}$ denotes the average retail prices of SLT per oz in state *s* and the first year that the state imposed specific taxes. The rationale for using average prices from the first year was to reduce the influence of time-varying factors, such as state-level tax changes, tax pass-through rate changes, etc., on SLT prices, so that the tax incidence remained consistent if there were no tax changes. For states that imposed *ad valorem* taxes based on retail prices, we used the following formula to calculate the tax incidence:

$${Tax\;incidence}_{s,t}=\frac{\frac{{Price\_r}_{s,t}}{1+T_{s,t}}\times T_{s,t}}{{Price\_r}_{s,t}}=\frac{T_{s,t}}{1+T_{s,t}}$$

where ${Price\_r}_{s,t}$ denotes the retail prices of SLT in state *s* at time *t*, and $T_{s,t}$ denotes the *ad valorem* tax rates at the retail level for state *s* at time *t*. Since Nielsen prices include excise taxes but exclude sales taxes, the numerator in the first step represents the total taxes paid. For states that imposed *ad valorem* taxes based on wholesale prices, we used the following formula to calculate the tax incidence:

$${Tax\;incidence}_{s,t}=\frac{{Price\_w}_{s,t}\times T_{s,t}}{{Price\_r}_{s,t}}$$

where ${Price\_w}_{s,t}$ denotes the wholesale prices of SLT in state *s* at time *t*, and $T_{s,t}$ denotes the *ad valorem* tax rates at the wholesale level for state *s* at time *t*. Similarly, the numerator represents the total taxes paid. We used the following formula to calculate the wholesale prices based on Nielsen retail prices:

$$\left({Price\_w}_{s,t}+{Price\_w}_{s,t}\times T_{s,t}\right)\times (1+m)={Price\_r}_{s,t}$$

where *m* denotes the markup rate from wholesale to retail prices. Therefore, the tax incidence was calculated as follows:

$${Tax\;incidence}_{s,t}=\frac{{Price\_w}_{s,t}\times T_{s,t}}{{Price\_r}_{s,t}}=\frac{{Price\_r}_{s,t}}{\left(1+T_{s,t}\right)\times (1+m)}\times \frac{T_{s,t}}{{Price\_r}_{s,t}}=\frac{T_{s,t}}{\left(1+T_{s,t}\right)\times (1+m)}$$

For states that imposed *ad valorem* taxes based on manufacturer prices, we used the following formula to calculate the tax incidence:

$${Tax\;incidence}_{s,t}=\frac{{Price\_m}_{s,t}\times T_{s,t}}{{Price\_r}_{s,t}}$$

where ${Price\_m}_{s,t}$ denotes the manufacturer prices of SLT in state *s* at time *t*, and $T_{s,t}$ denotes the *ad valorem* tax rates at the manufacturer level for state *s* at time *t*. Again, the numerator represents the total taxes paid. We used the following formula to calculate the manufacturer prices based on Nielsen retail prices:

$$\left({Price\_m}_{s,t}+{Price\_m}_{s,t}\times T_{s,t}\right)\times (1+m)={Price\_w}_{s,t}\;{Price\_w}_{s,t}\times (1+m)={Price\_r}_{s,t}\;\;\;\;\;\;\;\;$$

where *m* denotes the markup rate from manufacturer to wholesale prices and from wholesale to retail prices. Therefore, the tax incidence was calculated as follows:

$${Tax\;incidence}_{s,t}=\frac{{Price\_m}_{s,t}\times T_{s,t}}{{Price\_r}_{s,t}}=\frac{T_{s,t}}{\left(1+T_{s,t}\right)\times (1+m)\times (1+m)}$$

We followed previous literature and used a 20% markup rate at each distribution level [25].

#### 2.3.2. Examining the Tax Pass-Through Rate

We merged SLT tax data with the NRSD at the quarterly level, resulting in a state-by-quarter panel dataset between 2006 and 2020. Then, we implemented the ordinary least square (OLS) method within a fixed-effect (FE) framework in the benchmark model to examine how specific taxes are passed through prices. The specification of the model can be described as follows:

(1) $$P_{sij}=\alpha_{1}+\alpha_{2}{Tax}_{sij}{+X_{sij}\gamma +Y}_{i}+Q_{j}+\varphi_{s}+\varepsilon_{sij}$$

where $P_{sij}$ denotes SLT prices at the 25th, 50th, or 75th percentile. ${Tax}_{sij}\;$ denotes total excise taxes that include the federal- and state-specific taxes in state *s*, year *i*, and quarter *j*, respectively. $X_{sij}$ denotes state-level tobacco control policy variables, such as smoking and vaping bans in private worksites, restaurants, and bars, and MLSA laws for cigarettes and ECs. $Y_{i}$, $Q_{j}$, and $\varphi_{s}$ denote year-, quarter-, and state-fixed effects, respectively. $\varepsilon_{sij}$ is the error term. The fixed-effects model can account for time-invariant state-specific unobservable factors and common trends across states. Thereby, the association between taxes and prices could have a causal interpretation. Standard errors were clustered at the state level to account for inter-temporal correlations within each state.

Considering that EC use prevalence started to soar in 2012 and that nicotine salt ECs started to grow significantly in 2017, we then tested the difference in tax pass-through rates by different periods: the pre-EC uptake era (2006–2011), the EC uptake era (2012–2016), and the evolution of nicotine salt-based ECs era (2017–2020), to ascertain whether the tax pass-through rate was impacted by the uptake and evolution of ECs in the tobacco market. Specifically, we added two interaction terms between taxes and the EC uptake indicator (coded as 1 from 2012 onward and 0 before 2012) and the EC evolution indicator (coded as 1 from 2017 onward and 0 before 2017) to the estimating equation. The specification of the more comprehensive model can be described as follows:

(2) $$P_{sij}=\alpha_{1}+\alpha_{2}{Tax}_{sij}+{\alpha_{3}{Tax}_{sij}\times {EC\_uptake}_{sij}+\alpha_{4}{Tax}_{sij}\times {EC\_evolution}_{sij}+X_{sij}\gamma +Y}_{i}+Q_{j}+\varphi_{s}+\varepsilon_{sij}$$

Throughout the analysis, we also conducted seemingly unrelated regressions (SUR) to simultaneously estimate equations for SLT prices at different price levels. SUR allows the error terms of different estimating equations to be correlated, further allowing for a direct test of whether the estimated coefficients from different equations are statistically significant from each other. To be in line with existing literature, we classified tax pass-through rates into under-shifting (rate < 0.9), fully shifting (rate = 0.9–1.1), and over-shifting (rate > 1.1) [26,27]. Under-shifting implies that less than 90% of the tax burden is passed on to consumers through increased prices. Fully shifting implies that between 90% and 110% of the tax is transferred to consumers via higher prices. Finally, over-shifting indicates that more than 110% of the tax is shifted onto consumers through increased prices.

## 3. Results

**Figure 1:**
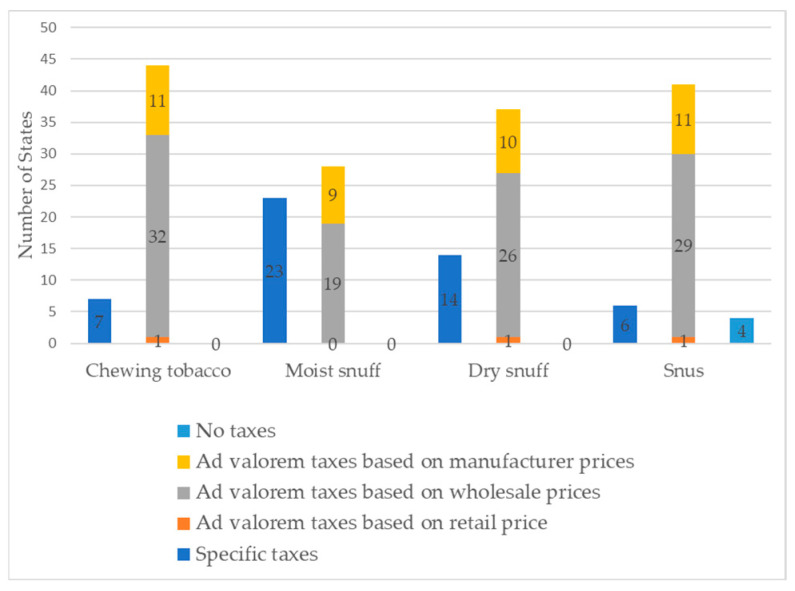
Tax schemas of SLT as of December 2020.

Table 1 shows the tax schemas of SLT in the US as of December 2020. Among all the states, 7 imposed specific taxes and 44 imposed *ad valorem* taxes on chewing tobacco. For moist snuff, the numbers were 23 for specific taxes and 28 for *ad valorem* taxes. As for dry snuff, 14 states applied specific taxes, while 37 applied *ad valorem* taxes. Regarding snus, 6 states had specific taxes, and 41 had *ad valorem* taxes. Notably, four states did not impose any taxes on snus. Among states that imposed *ad valorem* taxes, the tax bases varied (see Figure 1).

**Figure 2:**
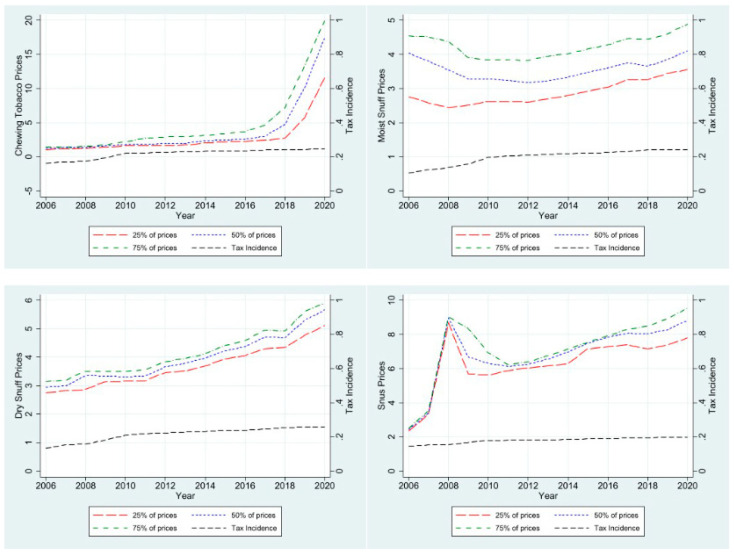
Trend for SLT prices and state-level tax incidence, 2006-2020.

Figure 2 exhibits the trend for real prices and tax incidence for each type of SLT from 2006 to 2020. The price trend for chewing tobacco was relatively flat from 2006 to 2017, followed by a notable surge between 2017 and 2020 (*p* < 0.05). In contrast, the tax incidence exhibited a steadily increasing trend, ranging from 16.3% in 2006 to 24.4% in 2020 (*p* < 0.001). For moist snuff, the price trajectory decreased from 2006 to 2009, subsequently transitioning into a gradual uptrend, which persisted through 2020 (*p* < 0.01). Meanwhile, the tax incidence for moist snuff showed an initial ascent from 2006 to 2010 and remained relatively stable for the remainder of the study period (*p* < 0.001). The prices of dry snuff demonstrated an upward trajectory throughout the entire study period (*p* < 0.001). However, the tax incidence of dry snuff exhibited a steadily increasing pattern over the same period (*p* < 0.001). The lowest tax incidence occurred in 2006 at 13.4%, while the highest occurred in 2020 at 25.9%. The price dynamics of snus were marked by a significant increase from 2006 to 2008, followed by a substantial decrease from 2008 to 2010. Beyond 2011, the prices for snus resumed an upward trajectory until 2020 (*p* < 0.01). In contrast, the tax incidence on snus steadily increased throughout the study period, ranging from 14.5% in 2006 to 19.7% in 2020 (*p* < 0.001).

Table 2 presents the summary statistics of tax incidence for all samples and by tax category. The average tax incidence of chewing tobacco, moist snuff, dry snuff, and snus for all samples during the study period was 22.2%, 21.5%, 22.8%, and 19.9%, respectively. The tax incidence for chewing tobacco, moist snuff, and dry snuff was higher in states that imposed specific taxes, compared to states that imposed *ad valorem* taxes. Conversely, the tax incidence for snus was higher in states that imposed *ad valorem* taxes, compared to states that imposed specific taxes. The two-sample *t*-test showed that the difference in tax incidence by tax form for all types of SLT was statistically significant.

In examining how specific taxes on SLT impact prices across states, we conducted three separate regressions for each type of SLT by price levels (the 25th, 50th, and 75th percentile prices). Table 3 provides summary statistics for the analytical sample. Among states with specific taxes, the average specific tax per ounce for chewing tobacco, moist snuff, dry snuff, and snus during the study period was USD 0.56, 0.95, 0.60, and 1.13, respectively.

Table 4 presents tax pass-through results using regressions with and without interaction terms between taxes and time period indicators for EC uptake and revolution. We found that for chewing tobacco, tax pass-through rates to prices were not significant across all price levels, regardless of the presence of the interaction terms. However, for moist snuff, taxes were fully passed to prices at the 25th and 50th percentiles (rate = 1.1, *p* < 0.001) and overly passed to prices at the 75th percentile (rate = 1.25, *p* < 0.001). The EC uptake and evolution did not significantly impact the tax pass-through rate for moist snuff at the 25th and 50th percentile prices. In contrast, at the 75th percentile prices, the EC uptake and evolution significantly raised taxes by 13 cents and 14 cents per ounce, respectively, for moist snuff (*p* < 0.05). The SUR test showed that the tax pass-through rates to prices for moist snuff at the 25th and 50th percentile were not statistically different. However, the tax pass-through rate at the 75th percentile price was significantly higher than that at the 25th and 50th percentile prices (*p* < 0.01).

For dry snuff, tax pass-through rates to prices were positive but not significant before controlling for interaction terms. However, after accounting for interaction terms, taxes were overly shifted to prices for dry snuff at all price levels. The EC uptake and evolution did not significantly affect tax pass-through rates to prices. For snus, taxes were overly passed to prices at all levels. However, the tax pass-through rate for snus at the 25th percentile prices almost doubled that at the 75th percentile prices. The EC uptake increased taxes by 80 cents per ounce of snus at the 50th percentile prices. The SUR test showed that tax pass-through rates to prices for chewing tobacco and dry snuff at different price levels were not statistically different. In contrast, for snus, the test showed that the tax pass-through rates at the 25th and 50th percentile prices were not statistically different, but the tax pass-through rate at the 75th percentile price was significantly lower than that at the 25th and 50th percentile prices (*p* < 0.05).

## 4. Discussion and Conclusions

Though states in the US have been taxing SLT to curb its use for several decades, the tax incidence of each type of SLT has not been thoroughly studied due to the complexity of tax schemas. This study marks the first attempt to calculate the tax incidence for each type of SLT product in the US, offering valuable evidence for policymakers to refine the taxation framework for SLT. In addition, the nicotine and tobacco marketplace has evolved significantly in the past two decades, with ECs and other emerging products entering the market and the merging and acquisition of nicotine and tobacco manufacturers. Policymakers have increasingly focused on how to tax nicotine and tobacco products relative to their harms [28,29]. Therefore, it is essential to assess the existing tax incidence and pass-through rates for SLT, which may be less harmful than cigarettes but still lead to adverse health consequences and attract consumers, especially among those who live in rural places or have low socioeconomic status (SES).

Our analysis showed that, between 2006 and 2020, the national average tax incidence on SLT ranged from 20% to 23%. To compare with the tax incidence on other tobacco products previously published by Shang et al. [13], we further calculated the tax incidence in 2020. Our results showed that the national average tax incidence on chewing tobacco, moist snuff, dry snuff, and suns in 2020 was 24.7%, 25.6%, 26.7%, and 19.9%, respectively, which was comparable to the 22% and 27% tax incidence on closed-system electronic nicotine delivery systems (ENDS) and open-system ENDS, but notably lower than the 42% tax incidence on cigarettes [13]. In addition, the tax incidence was very similar across various SLT types, despite the different tax schemes of these types. In other words, the cigarette tax incidence was almost twice as high as the SLT tax incidence. Considering the harms of SLT, the tax incidence on SLT may need to be further increased, especially as other alternative products like ENDS bear a higher tax incidence than SLT [29].

Our investigation also revealed that the state-level tax incidence on chewing tobacco, moist snuff, and dry snuff tended to be higher in states employing specific taxes compared to those utilizing *ad valorem* taxes. This suggests that specific taxes may have a more significant impact on increasing prices, aligning with findings in prior research [30]. However, a contrasting pattern emerged for snus, where the tax incidence was higher in states employing *ad valorem* taxes. Nonetheless, considering that moist snuff accounts for the majority of the SLT market, with a market share of nearly 91% [8], it is, therefore, safe to conclude that, on average, specific taxes on SLT are associated with higher tax incidence than *ad valorem* taxes. Consequently, if there is a future consideration to further elevate the overall SLT excise tax rate, specific taxes based on weights are preferred to value- or price-based *ad valorem* taxes.

We further examined tax pass-through rates in states imposing specific taxes for each SLT type. Caution is advised in interpreting the results for chewing tobacco, dry snuff, and snus due to the relatively small sample sizes and market shares. Nevertheless, the tax pass-through rates for all SLT types except chewing tobacco suggested full- or over-shifting of taxes to prices. These results align with established evidence in previous literature on tax pass-through rates for cigarettes and ECs [14,15,17,31,32,33].

In the case of moist snuff, the dominant SLT type, our findings indicated a full pass-through of taxes to prices at the 25th and 50th percentile levels, with an excessive pass-through observed at the 75th percentile level. This suggests that the tax pass-through rate is higher for higher-priced moist snuff. It implies that manufacturers strategically aim to derive profits from premium SLT while maintaining lower prices for budget or discount SLT [32,34]. Considering that individuals with lower SES are more likely to engage in price-minimizing behaviors, increasing tax pass-through rates for lower-priced SLT could effectively eliminate inexpensive products and encourage cessation [35]. This finding closely parallels those of He et al. (2023), who found that cigarette excise taxes were fully shifted to the 25th and 50th percentile prices and overly shifted to the 75th percentile prices at a 1 to 1.1 rate [14]. This suggests that the tobacco industry is adopting similar pricing strategies. This is not surprising considering that US cigarette companies not only purchase SLT companies but also manufacture their own SLT brands [20].

Moreover, the uptake and evolution of ECs did not significantly impact moist snuff at the 25th and 50th percentile prices. However, at the 75th percentile prices, they elevated tax pass-through rates by 13 and 14 cents for moist snuff, respectively. This again is similar to the findings for cigarette tax pass-through rates at 75th percentile prices during the uptake and evolution of ECs [14]. It is possible that due to the competition of ECs, tobacco companies raised tax pass-through rates for high-priced SLTs to increase profits [20,32,34].

Determining tax pass-through rates is critical in assessing the effectiveness of tax policies in influencing behaviors [14,36,37]. Through comparisons with tax pass-through rates for other tobacco products, it becomes evident that increasing taxes on moist snuff, particularly higher-priced variants, can have a meaningful impact on reducing use. Considering the lower tax pass-through rates for lower-priced moist snuff, it is imperative to sustain efforts to raise taxes, complemented by additional pricing policies, such as restrictions on price promotions. These measures are essential to raise retail prices and curtail opportunities for price minimization.

Though our study is the first to examine the tax incidence and tax pass-through rates of SLT, it is not exempt from certain limitations. First, we used arbitrary cutoffs to classify under-shifting, fully shifting, and over-shifting of taxes to prices, a method derived from existing literature. However, our findings and the classification of tax pass-through rates remained largely consistent when utilizing a single cutoff (e.g., considering rates > 1 as over-shifting and <1 as under-shifting). Second, the EC uptake and evolution timeline may differ by state, potentially introducing variability that could impact the interpretation of our results. Nevertheless, this timeline aligns with the broader development of the EC market and has been utilized in previously published articles [14]. In recognizing these limitations, our study sets the foundation for further investigations that can refine methodologies and deepen our understanding of the complexities of SLT taxation and evolving market landscapes.

In summary, if harm reduction is a guiding criterion for taxing tobacco products, the tax incidence on SLT products could be raised to reflect their associated health risks more effectively. Our findings showed that lower-priced SLT products have lower tax pass-through rates, suggesting that price promotion restrictions and minimum pricing laws may be necessary to reduce the affordability of these products and discourage consumption. Additionally, we observed that tobacco companies tended to increase tax pass-through for premium SLT products as ECs gained popularity, which may indicate a strategic response to shifting consumer preferences. Future research could explore the long-term impact of price promotion restrictions and minimum pricing laws on SLT consumption patterns and public health outcomes. It would also be valuable to investigate how these policies influence consumer substitution between SLT and other tobacco products, such as ECs.

## Figures and Tables

**Table 1:** Tax schemas of SLT as of December 2020.

Type of SLT	Tax Schema	Number	States
Chewing tobacco	Specific taxes	7	AL, AZ, KY, ME, ND, PA, TX
	*Ad valorem* taxes	44	1 based on retail prices: WA 32 based on wholesale prices: AK, CA, CT, DC, DE, FL, GA, HI, IA, ID, IL, IN, KS, MA, MD, MI, MN, MT, NC, NE, NH, NJ, NV, NY, OH, OR, RI, SD, TN, VT, WV, WY 11 based on manufacturer prices: AR, CO, LA, MO, MS, NM, OK, SC, UT, VA, WI
	No taxes	0	
Moist snuff	Specific taxes	23	AL, AZ, CT, DE, IA, IL, IN, KY, ME, MT, ND, NE, NJ, NY, OR, PA, RI, TX, UT, VA, VT, WA, WY
	*Ad valorem* taxes	28	19 based on wholesale prices: AK, CA, DC, FL, GA, HI, ID, KS, MA, MD, MI, MN, NC, NH, NV, OH, SD, TN, WV 9 based on manufacturer prices: AR, CO, LA, MO, MS, NM, OK, SC, WI
	No taxes	0	
Dry snuff	Specific taxes	14	AL, AZ, CT, IA, KY, ME, ND, NE, NY, PA, RI, TX, VA, VT
	*Ad valorem* taxes	37	1 based on retail prices: WA 26 based on wholesale prices: AK, CA, DC, DE, FL, GA, HI, ID, IL, IN, KS, MA, MD, MI, MN, MT, NC, NH, NJ, NV, OH, OR, SD, TN, WV, WY 10 based on manufacturer prices: AR, CO, LA, MO, MS, NM, OK, SC, UT, WI
	No taxes	0	
Snus	Specific taxes	6	AZ, KY, ME, PA, TX, VT
	*Ad valorem* taxes	41	1 based on retail prices: WA 29 based on wholesale prices: AK, CA, DC, DE, FL, GA, HI, IA, ID, IL, IN, KS, MA, MD, MI, MN, MT, NC, NE, NJ, NV, NY, OH, OR, RI, SD, TN, WV, WY 11 based on manufacturer prices: AR, CO, LA, MO, MS, NM, OK, SC, UT, VA, WI
	No taxes	4	AL, CT, ND, NH

**Table 2:** Summary statistics of the state-level tax incidence, 2006–2020.

SLT Type	All Samples	States That Imposed Specific Taxes		States That Imposed *Ad Valorem* Taxes		*p*-Value
	Mean (SD)	# of obs.	Mean (SD)	# of obs.	Mean (SD)	
Chewing tobacco	0.222 (0.149)	339	0.292 (0.285)	2663	0.213 (0.119)	<0.001
Moist snuff	0.215 (0.144)	1164	0.238 (0.168)	1658	0.199 (0.123)	<0.001
Dry snuff	0.228 (0.159)	710	0.293 (0.230)	2172	0.207 (0.121)	<0.001
Snus	0.199 (0.111)	267	0.170 (0.113)	2483	0.202 (0.110)	<0.001

**Table 3:** Summary statistics of the analytical sample, 2006–2020.

	Chewing Tobacco Mean (SD)	Moist Snuff Mean (SD)	Dry Snuff Mean (SD)	Snus Mean (SD)
Outcome variables				
SLT prices at 25-percentile	3.035 (3.980)	3.315 (1.005)	3.911 (1.142)	7.616 (2.494)
SLT prices at 50-percentile	4.184 (5.482)	3.855 (0.976)	4.239 (1.257)	7.994 (2.412)
SLT prices at 75-percentile	5.269 (5.978)	4.380 (0.939)	4.426 (1.280)	8.360 (2.436)
Explanatory variables				
Specific tax	0.562 (0.634)	0.948 (0.650)	0.600 (0.466)	1.129 (0.771)
State-level tobacco control policy variables				
Smoking bans in private worksites	0.584 (0.494)	0.708 (0.455)	0.357 (0.480)	0.693 (0.462)
Smoking bans in private restaurants	0.451 (0.498)	0.625 (0.484)	0.289 (0.454)	0.622 (0.486)
Smoking bans in private bars	0.451 (0.498)	0.669 (0.471)	0.289 (0.454)	0.622 (0.486)
Vaping bans in private worksites	0.097 (0.297)	0.165 (0.371)	0.003 (0.057)	0.072 (0.259)
Vaping bans in private restaurants	0.159 (0.366)	0.183 (0.387)	0.003 (0.057)	0.155 (0.363)
Vaping bans in private bars	0.159 (0.366)	0.183 (0.387)	0.003 (0.057)	0.155 (0.363)
Minimum legal sales age (MLSA) laws for cigarettes	0.018 (0.132)	0.069 (0.253)	0.049 (0.217)	0.048 (0.214)
Minimum legal sales age (MLSA) laws for e-cigarettes	0.018 (0.132)	0.069 (0.253)	0.049 (0.217)	0.048 (0.214)
# of obs.	339	1164	305	251

**Table 4:** SLT tax pass-through rates, 2006–2020.

Control Variables	SLT Prices at 25%		SLT Prices at 50%		SLT Prices at 75%	
Chewing Tobacco (n = 339)						
Standardized SLT excise tax	−2.519 (0.880)	−15.981 (0.318)	−0.555 (0.924)	−6.043 (0.336)	−10.535 (0.250)	−11.210 (0.280)
SLT tax × EC uptake		−0.795 (0.365)		−0.381 (0.296)		−0.585 (0.140)
SLT tax × EC evolution		−1.828 (0.182)		−0.680 (0.323)		0.533 (0.452)
R^2^	0.646	0.653	0.836	0.836	0.868	0.869
Pass-through rates						
2006–2011	--	−15.981 (0.318)	--	−6.043 (0.336)	--	−11.210 (0.280)
2012–2016	--	−16.776 (0.282)	--	−6.424 (0.289)	--	−11.794 (0.230)
2017 and later	--	−18.604 (0.205)	--	−7.103 (0.242)	--	−11.262 (0.270)
Moist Snuff (n = 1164)						
Standardized SLT excise tax	1.014 *** (<0.001)	0.882 *** (<0.001)	1.011 *** (<0.001)	0.920 *** (<0.001)	1.247 *** (<0.001)	1.041 *** (<0.001)
SLT tax × EC uptake		0.155 (0.236)		0.071 (0.528)		0.131 * (0.017)
SLT tax × EC evolution		−0.005 (0.952)		0.044 (0.466)		0.138 * (0.043)
R^2^	0.924	0.925	0.933	0.934	0.949	0.953
Pass-through rates						
2006–2011	--	0.882 *** (<0.001)	--	0.920 *** (<0.001)	--	1.041 *** (<0.001)
2012–2016	--	1.037 *** (<0.001)	--	0.991 *** (<0.001)	--	1.172 *** (<0.001)
2017 and later	--	1.032 *** (<0.001)	--	1.035 *** (<0.001)	--	1.310 *** (<0.001)
Dry Snuff (n = 305)						
Standardized SLT excise tax	0.829 (0.240)	1.314 * (0.029)	2.664 (0.113)	1.762 * (0.049)	2.818 (0.075)	1.8440* (0.018)
SLT tax × EC uptake		−0.037 (0.946)		−0.160 (0.807)		−0.011 (0.484)
SLT tax × EC evolution		0.303 (0.538)		−0.393 (0.605)		−0.545 (0.619)
R^2^	0.798	0.800	0.776	0.780	0.801	0.807
Pass-through rates						
2006–2011	--	1.314 * (0.029)	--	1.762 * (0.049)	--	1.844 * (0.018)
2012–2016	--	1.276 (0.091)	--	1.603 (0.159)	--	1.833 * (0.030)
2017 and later	--	1.580 ** (0.002)	--	1.210 (0.114)	--	1.287 * (0.034)
Snus (n = 251)						
Standardized SLT excise tax	4.341 *** (<0.001)	4.131 *** (<0.001)	4.478 ** (0.001)	3.495 *** (<0.001)	2.130 *** (<0.001)	2.301 *** (<0.001)
SLT tax × EC uptake		0.186 (0.200)		0.795 * (0.044)		−0.140 (0.370)
SLT tax × EC evolution		0.135 (0.390)		0.152 (0.404)		−0.034 (0.610)
R^2^	0.924	0.924	0.906	0.913	0.961	0.961
Pass-through rates						
2006–2011	--	4.131 *** (<0.001)	--	3.495 *** (<0.001)	--	2.301 *** (<0.001)
2012–2016	--	4.318 *** (<0.001)	--	4.289 *** (<0.001)	--	2.161 *** (<0.001)
2017 and later	--	4.453 *** (<0.001)	--	4.441 *** (<0.001)	--	2.127 *** (<0.001)

Note: * *p* < 0.05, ** *p* < 0.01, and *** *p* < 0.001. State-level tobacco control policies include smoking and vaping bans in private worksites, restaurants, and bars for both cigarettes and e-cigarettes, as well as minimum legal sales age (MLSA) laws for cigarettes and e-cigarettes.

## Data Availability

The datasets generated and/or analyzed during this study are available from the corresponding author upon reasonable request.
